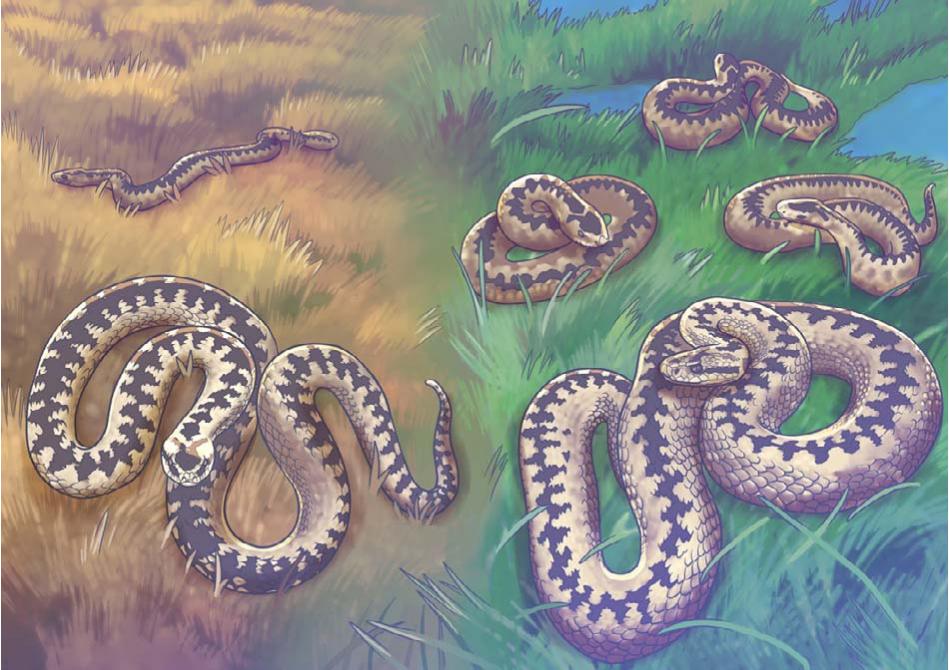# Momma needs a drink: droughts are stressing out pregnant snakes

**DOI:** 10.1093/conphys/coac007

**Published:** 2022-02-22

**Authors:** Daniel F Gomez Isaza

**Affiliations:** School of Biological Science, The University of Queensland, Brisbane, Queensland 4072, Australia

Mothers invest a lot of time into the healthy development of their young. Yet, climate change is making motherhood more challenging. Water and nutrients are becoming scarce. Indeed, a recent study, led by Mathias Dezetter from the Centre for Biological Studies of Chizé in France, found that snakes are no exception. Populations of European adder (*Vipera berus*) may ‘dry out’ if drought conditions worsen.

Heatwaves and droughts are becoming increasingly common with climate change. Periods of drought can pose physiological challenges to animals. Even a few days without water can increase the risk of extreme physiological stress if not lethal dehydration. Drought conditions are particularly tricky for pregnant female snakes because they sacrifice considerable amounts of water to support the development of their embryos. This forces an internal water conflict—either support the growth and development of their offspring or their own self-maintenance.

While snakes are not known for their ‘motherly instincts’, the European adder is an exception. European adders give birth to live young instead of laying eggs. Pregnant adders carry their young for up to four gruelling summer months; during which time, they must find limited resources for both themselves and their developing embryos. Unfortunately for these snakes, they now face longer periods without water. This led Dezetter and his team to wonder about the future stability of European adder populations as they struggle to reproduce amidst the drying landscape.

Dezetter and colleagues captured pregnant adders and exposed them to simulated summer drought conditions for 15 days. The team took measurements of body mass and tail width as indicators of condition during drought exposure. Small blood samples were also taken from snakes to assess their hydration status (plasma osmolality) and stress levels. The researchers used high-resolution ultrasonography (i.e. ultrasound) to get a ‘sneak peek’ into how the developing embryos were coping with drought conditions. Not only could they track embryo development, but they also could differentiate between live and dead embryos by the presence of tiny heartbeats and slithery movements.

The team found that drought conditions took a massive toll on pregnant European adders. Drought-exposed snakes were severely dehydrated, as indicated by a spike in plasma osmolality. The condition of pregnant adders also plummeted. During the short, 15-day simulated drought, mother snakes lost a staggering 9% of their body mass and their tail muscle mass deteriorated as well. This loss of body condition was worse in females carrying more embryos, indicating a strong parent–offspring conflict for water during periods of drought. Dezetter and colleagues also noted exceptionally high levels of the stress hormone, corticosterone, in adders living in drought conditions, which resulted in high levels of embryonic mortality.

Dezetter and colleagues’ findings suggest that drought-induced population declines may be in store for the European adder unless we enact conservation measures. Drought conditions are expected to worsen with climate change, which will make pregnancy in European adders even more expensive. Snakes may bear fewer young and suffer a high chance of mortality after pregnancy, as a result. Management and conservation projects can harness reliable biological indicators of stress and hydration status to identify individuals and populations of the European adder that are at risk of drought-induced mortality. Conserving wetlands and other humid environments will also be paramount; such habitats can serve as refuge areas for the European adder and other drought-sensitive species.

**Illustrations**: Erin Walsh, ewalsh.sci@gmail.com